# Nuclear lamina component KAKU4 regulates chromatin states and transcriptional regulation in the *Arabidopsis* genome

**DOI:** 10.1186/s12915-024-01882-5

**Published:** 2024-04-12

**Authors:** Yaxin Cao, Hengyu Yan, Minghao Sheng, Yue Liu, Xinyue Yu, Zhongqiu Li, Wenying Xu, Zhen Su

**Affiliations:** State Key Laboratory of Plant Environmental Resilience, College of Biological Sciences, China Agricultural University, Beijing, 100193 China

**Keywords:** *Arabidopsis*, KAKU4, Chromatin state, H3K27me3, H3K9me2, Hormone pathway

## Abstract

**Background:**

The nuclear lamina links the nuclear membrane to chromosomes and plays a crucial role in regulating chromatin states and gene expression. However, current knowledge of nuclear lamina in plants is limited compared to animals and humans.

**Results:**

This study mainly focused on elucidating the mechanism through which the putative nuclear lamina component protein KAKU4 regulates chromatin states and gene expression in *Arabidopsis* leaves. Thus, we constructed a network using the association proteins of lamin-like proteins, revealing that KAKU4 is strongly associated with chromatin or epigenetic modifiers. Then, we conducted ChIP-seq technology to generate global epigenomic profiles of H3K4me3, H3K27me3, and H3K9me2 in *Arabidopsis* leaves for mutant (*kaku4-2*) and wild-type (WT) plants alongside RNA-seq method to generate gene expression profiles. The comprehensive chromatin state-based analyses indicate that the knockdown of *KAKU4* has the strongest effect on H3K27me3, followed by H3K9me2, and the least impact on H3K4me3, leading to significant changes in chromatin states in the *Arabidopsis* genome. We discovered that the knockdown of the *KAKU4* gene caused a transition between two types of repressive epigenetics marks, H3K9me2 and H3K27me3, in some specific PLAD regions. The combination analyses of epigenomic and transcriptomic data between the *kaku4-2* mutant and WT suggested that KAKU4 may regulate key biological processes, such as programmed cell death and hormone signaling pathways, by affecting H3K27me3 modification in *Arabidopsis* leaves.

**Conclusions:**

In summary, our results indicated that KAKU4 is directly and/or indirectly associated with chromatin/epigenetic modifiers and demonstrated the essential roles of KAKU4 in regulating chromatin states, transcriptional regulation, and diverse biological processes in *Arabidopsis*.

**Supplementary Information:**

The online version contains supplementary material available at 10.1186/s12915-024-01882-5.

## Background

The nuclear lamina (NL), a vital component of the nuclear membrane, is located on the inner surface of the nuclear envelope. It functions as a structural component, maintaining the integrity of the nucleus and also providing anchoring sites for chromatin domains, signaling molecules, and transcription factors to support a variety of cellular functions [3, 42, 68, 84, 94]. Lamins form filaments constituting the scaffold of nuclear lamina and play fundamental functions in nuclear mechanics and genome regulation [16, 18, 31, 83]. Defects in lamin genes can affect the morphology of the nucleus and lead to the occurrence of human lamin-associated diseases, including Hutchinson-Gilford progeria syndrome, heart-hand syndrome, and Alzheimer’s disease [15, 20, 60]. Lamins can respond to environmental and developmental cues by adjusting their localization, interaction partners, and eventually epigenetic landscapes and gene regulatory networks.

No lamin-homologous proteins are found in plants, although some putative lamina-like structures have been previously discovered. The nuclear matrix constituent protein (NMCP1) was first identified as a peripheral framework component of its nuclei [54]. Currently, lamin-like proteins in plants include CRWNs [19, 66, 88], KAKU4 [25, 26], NEAPs [62], and PNET2 [77]. CRWNs were named because of crowded nuclei, and they are NMCP homologs, including CRWN1, CRWN2, CRWN3, and CRWN4 [88]. *Arabidopsis* CRWN1 and CRWN4 are localized to the nuclear periphery and are thought to act as functional analogues of lamins [12, 19, 66]. CRWN1 and CRWN4 play essential roles in maintaining the nuclear architecture, and their single mutants show reduced nuclear size, altered nuclear morphology, and disordered spatial heterochromatin organization [88]. CRWN2 is localized in the nucleoplasm, and CRWN3 is found in the nucleoplasm and nuclear periphery [66]. KAKU4 localizes to the inner nuclear membrane and modulates nuclear shape and size [25]. NEAPs and PNET2 also affect nuclear morphology [62, 77].

Nuclear lamina-chromatin interactions are key in maintaining the nuclear architecture. Chromatin is associated with the lamina, NPCs, and potentially other inner nuclear membrane components, while nuclear lamina is directly involved in chromatin tethering [27, 76]. In mammalian cells, up to one-third of the genome and 10% of coding genes are found within lamina-associated domains (LADs) [5, 45, 63], which are enriched with repressive histone modification, including H3K9me2, H3K9me3 and H3K27me3, and are sparsely populated with active chromatin marks, such as H3K4me [5, 34, 36, 81]. In *Arabidopsis*, CRWN1 and CRWN4 were found to be responsible for tethering specific pericentromeric chromatin regions to the nuclear periphery using high-throughput chromosome conformation capture (Hi-C) analysis and in situ hybridization assays. These non-accessible chromatin regions are termed plant lamina-associated domains (PLADs) and promote transcriptional repression of the genes. Losing either the *CRWN1* or *CRWN4* gene can alter the chromatin position patterns and attenuate chromatin compartmentalization [38]. Moreover, CRWN1 was recently identified to play a structural role in shaping the changes in genome folding under heat stress [89]. In addition, the interaction of AtNEAP1 with chromatin is suggested by the transcription factor, AtbZIP18, as an interaction partner and by altered localization of CFP-AtNEAP1 resulting from its co-expression with YFP-AtbZIP18 [62]. PNET2 mediates chromatin tethering to the nuclear periphery and is required to maintain the proper chromatin architecture and transcription programming [77].

A close relationship exists between nuclear lamina proteins and epigenetic marks. In mouse and human cells, the epigenetic marks (epi-marks) H3K9me2/H3K9me3 and H3K27me3 influence the frequency of NL-LAD interactions [42]. The constitutive heterochromatin marks H3K9me2 and H3K9me3 decorate the main bodies of the LADs, and the facultative heterochromatin mark H3K27me3 is enriched around the LAD boundaries. The abnormal function of nuclear lamina proteins often leads to changes in epi-marks H3K9me2/3 and H3K27me3. In addition, there is some evidence regarding the link between DNA methylation and nuclear lamina [6, 74]. A differentially methylated region (DMR) was identified at a distal region in the promoter of TERT (stem cell marker gene) between human iPSCs and their parental somatic cells. The transcription of TERT was enhanced by DNA methylation at ERT-DMR via binding to nuclear lamina during reprogramming [74]. In *Arabidopsis*, the nuclear lamina protein CRWN1 directly interacts with chromatin domains localized at the nuclear periphery, which are responsible for tethering specific pericentromeric chromatin regions to the nuclear periphery [38]. The epi-mark H3K9me2 is dispensable for chromatin tethering at the nuclear periphery. Meanwhile, CRWN1 mediates the epi-mark H3K27me3 histone modification through its direct physical interaction with PWO1.

The nuclear lamina component can interact with proteins in the nuclear periphery to perform the corresponding functions. Lamins often form complexes with nuclear proteins and play key roles in maintaining nuclear structure and gene regulation [31–33, 56]. Currently, there is an increasing amount of plant proteomic data related to the nuclear lamina and nuclear proteins in the nuclear periphery [39, 77–79]. The proximity labeling proteomics has become a powerful technique to identify the complex components among the interaction partners of CRWN1, KAKU4, PNET2_A/B, NEAP1, the nuclear basket protein GBPL3, and some other lamina-related components [39, 77–79]. CRWN1 shows direct physical interaction with the PWWP-domain interactor of polycombs1 (PWO1), a key component of polycomb repressive complex 2 (PRC2), which mediates H3K27me3 histone modification, the repressive chromatin mark, to induce transcriptional repression [37, 57, 64]. H3K27me3 accumulation levels near genes involved in SA biosynthesis decrease in *crwn* mutants [9, 10]. The GBPL3/CRWN1&4/KAKU4 complex is essential during interphase in H3K27me3-associated transcriptional repression at the nuclear periphery [65].

Although the knowledge about the structural incorporation of KAKU4 into the nuclear lamina is limited, KAKU4, as a unique nuclear lamina component in the nuclear periphery, physically interacts with CRWN1 and CRWN4 and modulates nuclear shape and size [25]. The mutants disrupted CRWN1, CRWN4, or KAKU4 expression display similar defects in nuclear morphogenesis [25, 66]. KAKU4 determines the migration order of the vegetative nucleus and sperm cells in pollen tubes possibly due to the nuclear shape in vegetative cells of pollen grains [26]. From the proximity labeling proteomics data, KAKU4 integrates into a common complex with CRWN1, CRWN4, GBPL3, and together with some epigenetic regulation factors, etc. [65, 77]. Therefore, it will be interesting to elucidate the potential functions of KAKU4 in regulating epigenetic landscapes and gene expression. The data presented in this study mainly demonstrate the important roles of KAKU4 in fine-tuning chromatin states and transcriptional regulation in *Arabidopsis* leaves.

## Results

### Protein-protein association network analysis revealed that KAKU4 may be related to chromatin or epigenetic modification

**Fig. 1:**
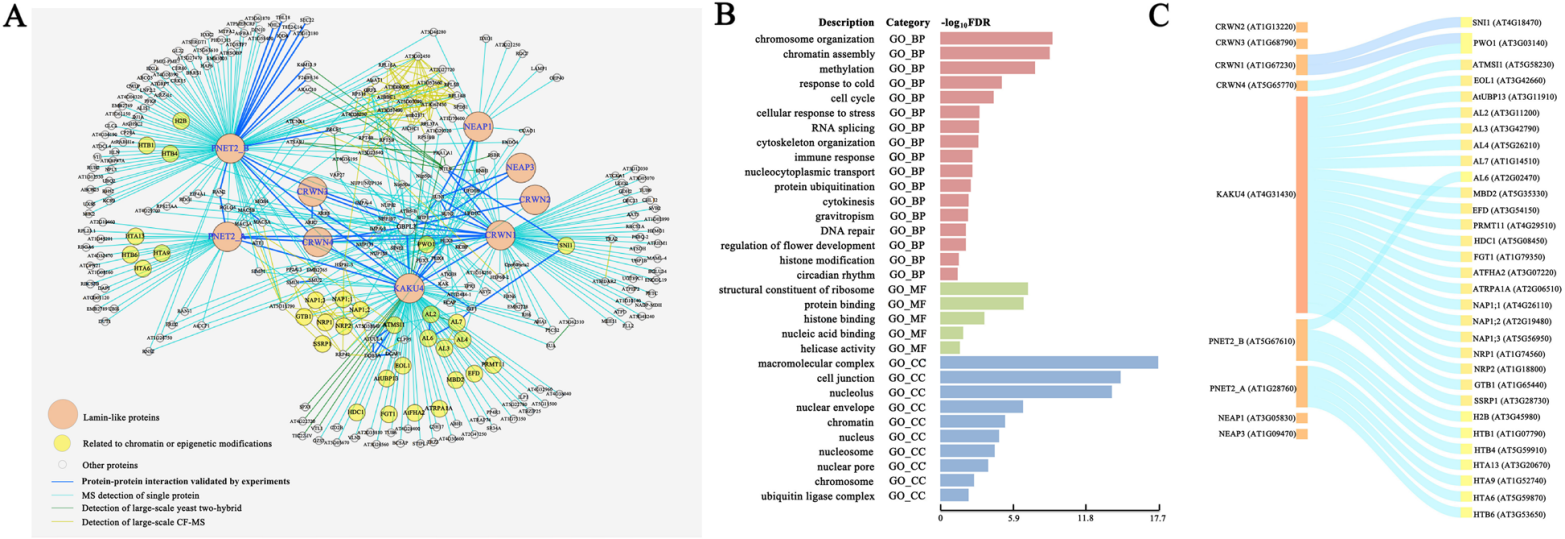
Protein-protein association network analysis of lamin-like proteins. **A** Protein-protein association network of lamin-like proteins. The orange nodes represent the lamin-like proteins; the yellow nodes represent the proteins related to chromatin or epigenetic modification; the gray nodes represent the other proteins. The line color represents the method of demonstrating protein-protein interactions: The dark blue line represents the experimental verification of protein-protein interaction, the light blue line represents MS verification of a single protein, the green line represents the identification of large-scale yeast two-hybrid, and the yellow line represents the identification of large-scale co-fractionation mass spectrometry (CF-MS). The thickness of the line represents the number of evidence. **B** GO enrichment analysis of lamin-like proteins and their possible associated proteins. The bar color represents the GO category: red indicates biological processes (BP), green indicates molecular functions (MF), and blue indicates cellular components (CC). **C** Chromatin or epigenetic modification proteins among associated proteins of lamin-like proteins

The nuclear lamina closely interacts with chromatin interactions and is vital in maintaining the nuclear architecture. Chromatin is associated with the lamina, NPCs, and potentially other inner nuclear membrane components, while the nuclear lamina is directly involved in chromatin tethering [27, 76]. We conducted text-mining in recent literature [39, 77–79] and the BioGRID database [61] to make a comprehensive integration of proteomic data related to lamin-like proteins in *Arabidopsis*. A protein-protein association network (Fig. 1A) was constructed using nine lamin-like proteins as hub nodes: CRWN1, CRWN2, CRWN3, CRWN4, KAKU4, NEAP1, NEAP3, PNET2_A, and PNET2_B. This network contains 283 nodes and 564 edges (Additional file 2: Table S1), with multiple protein interaction/association pairs between lamin-like proteins and the proteins related to nuclear envelope, nuclear pore complexes, chromatin, epigenetic modification, etc.

We performed gene set enrichment analysis (GSEA) through the PlantGSAD database [53] to infer the possible roles of the proteins in this network. The analysis result (Fig. 1B and Additional file 3: Table S2) shows that the enriched terms in different GSEA categories are mainly related to chromatin and epigenetic modification, such as “chromosome organization,” “chromatin assembly,” “histone modification,” “gene silencing,” and “DNA repair.” Meanwhile, some other terms were also enriched, which related to cell cycle, circadian, stress response, flower development, etc. Furthermore, we show the one-by-one association of individual lamin-like proteins to the proteins related to chromatin or epigenetic modification (Fig. 1C and Additional file 2: Table S1).

As a key node in the protein-protein association network (Fig. 1A and Additional file 2: Table S1), KAKU4 is closely associated with multiple lamin-like proteins (CRWN1, CRWN4, and PNET2_A ) and nuclear envelope-related proteins (such as SUN1, Nup50a, Nup50c, NUP82, NUP107, NUP133, NUP1/NUP136, GBPL3, IMPA-3, etc.), together with chromatin/epigenetic modification-related proteins, including PRC1-related proteins (Alfin-like proteins: AL2, AL3, AL4, and AL7), PRC2-related proteins (MSI1, EOL1, AtUBP13), DNA methylation-related proteins (MBD2, EFD and PRMT11), nucleosome assembly proteins (NAP1;1, NAP1;2, NAP1;3, NRP1, NRP2), and other proteins.

In summary, we constructed a protein-protein association network of lamin-like proteins, and network analysis initially revealed that KAKU4 may be associated with chromatin/epigenetic modification.

### Knockdown of *KAKU4* influenced the whole-genome distribution of H3K27me3 and H3K9me2

**Fig. 2:**
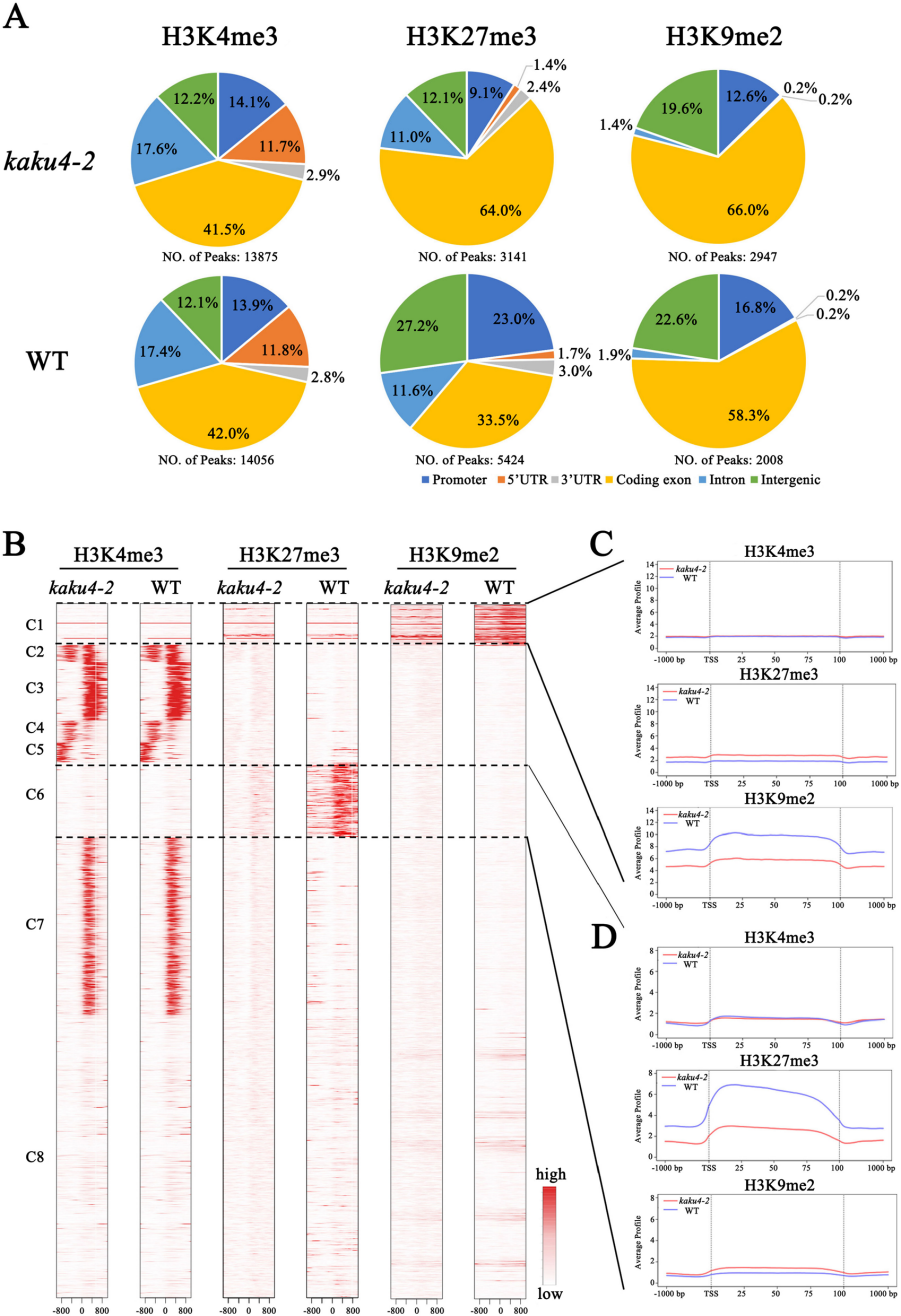
ChIP-seq analysis for histone modification in the *kaku4-2* mutant and WT. **A** The H3K4me3/H3K27me3/H3K9me2 distribution within different regions (promoter, 5′UTR, coding exon, intron, 3′UTR, and intergenic) of the *Arabidopsis* genome in *kaku4-2* mutant and WT. **B** The heatmaps display the H3K4me3/H3K27me3/H3K9me2 signal around TSSs in *kaku4-2* mutant and WT plants. For each gene, the H3K4me3/H3K27me3/H3K9me2 signals are displayed along −1 to 1 kb regions around the TSSs. C1–C8 are the eight parts after clustering. **C** Distribution of H3K4me3/H3K27me3/H3K9me2 along *Arabidopsis* genes in the C1 region. A meta-gene profile was generated using the normalized sequencing density of H3K4me3/H3K27me3/H3K9me2 in *kaku4-2* mutant and WT. The gene body was converted into a percentage to standardize genes of different lengths. The 1 kb upstream and downstream regions of the gene are included. **D** Distribution of H3K4me3/H3K27me3/H3K9me2 along *Arabidopsis* genes in the C6 region. A meta-gene profile was generated using the normalized sequencing density of H3K4me3/H3K27me3/H3K9me2 in *kaku4-2* mutant and WT

The protein-protein association network of the lamin-like proteins provides an important clue that KAKU4 may be associated with histone modification and chromatin state transition. Thus, we chose the typical active histone modification mark H3K4me3, the typical repressive histone modification mark H3K27me3, and the heterochromatin mark H3K9me2 to study how the mutation of KAKU4 influences histone methylation in the *Arabidopsis* genome. The *kaku4-2* (SALK_076754) was validated as a knockdown mutant by qRT-PCR experiment (Additional file 3: Table S14). We performed Western blot analysis to check if there is a potential interplay between KAKU4 and epigenetic marks. The results showed that the knockdown of *KAKU4* gene affected epigenetic marks H3K27me3 and H3K9me2 and had the least impact on H3K4me3 (Additional file 1: Fig S1). We further applied a high-throughput sequencing technique and generated global epigenomic profiles on *kaku4-2* mutant and WT to explore whether the knockdown of *KAKU4* affected the deposition of these three histone modifications. ChIP-seq analyses of H3K4me3, H3K27me3, and H3K9me2 were conducted using the 4th-week rosette leaves of the *kaku4-2* mutant and WT (Additional file 4: Table S3). The *Arabidopsis* genome was categorized into six genomic subregions: promoter, 5′ untranslated region (5′ UTR), 3′ UTR, exon, intron and intergenic region, based on the physical positions of the genes. Firstly, we characterized the distribution of the enriched peaks for H3K4me3, H3K27me3, and H3K9me2 of the *kaku4-2* mutant and WT in different genomic subregions (Fig. 2A). For H3K4me3, the identified peaks exhibited minimal difference between *kaku4-2* mutant and WT (13,875 vs. 14,056, more than 95% peaks were overlapped), as well as their distribution in different subregions of the genome (Fig. 2A and Additional file 4: Table S3). For H3K27me3, the identified peaks were significantly lower in the *kaku4-2* mutant than in the WT (3141 vs. 5424, a decrease of approximately 40%), and more than two-thirds of the peaks in the *kaku4-2* mutant were overlapped with those in the WT. Compared to the WT, the distribution of H3K27me3 deposition peaks was much higher in the coding exon region and lower in the promoter and intergenic regions in the *kaku4-2* mutant (Fig. 2A and Additional file 4: Table S3). Regarding H3K9me2, 2947 (more than 70% overlapped) and 2008 (more than 35% overlapped) enriched regions were identified in the *kaku4-2* mutant and WT, respectively. The peak distribution in the exon, promoter, and intergenic subregions showed a slight difference between the *kaku4-2* mutant and WT (Fig. 2A and Additional file 4: Table S3).

Then, we focused on the distribution of different histone modifications (H3K4me3, H3K27me3, and H3K9me2) around the TSS regions and clustered them into eight categories, named C1-C8 (Fig. 2B). Among them in the C1 cluster, the deposition of H3K9me2 was higher in WT plants than in the *kaku4-2* mutant; in the C6 cluster, the deposition of H3K27me3 was significantly higher in WT plants than in the *kaku4-2* mutant. By analyzing the meta-gene profiles in the gene body with the upstream and downstream 1-kb region, we found that in the C1 cluster, a higher level of H3K9me2 modification accumulated in the WT than the *kaku4-2* mutant (Fig. 2C), with a slightly lower level of H3K27me3 in the WT than the *kaku4-2* mutant. For the C6 cluster (Fig. 2D), significantly more H3K27me3 accumulated in the WT than in the *kaku4-2* mutant, whereas H3K9me2 was slightly lower in the WT than in the *kaku4-2* mutant. There was no significant change in H3K4me3 accumulation between the WT and *kaku4-2* in the C1 and C6 clusters.

We further identified the peaks and their associated genes with the differential deposition of H3K27me3, H3K9me2, and H3K4me3 between the *kaku4-2* mutant and WT (4-week-old *Arabidopsis* leaves). The knockdown of the *KAKU4* gene resulted in a significant change in the deposition of H3K27me3 in the genome. A total number of 2417 and 5510 enriched regions, associated with 2901 and 6349 genes, were identified with higher deposition in *kaku4-2* mutant and WT, respectively. We also found 595 and 1180 enriched regions (770 and 1265 genes) with higher H3K9me2 depositions in the *kaku4-2* mutant and WT, respectively. The knockdown of the *KAKU4* gene slightly influenced the genome distribution of H3K4me3; there were 120 and 166 enriched regions (124 and 223 genes) detected with differential depositions of H3K4me3 in the *kaku4-2* mutant and WT, respectively (Additional file 4: Table S4, Additional file 5: Table S5, and Additional file 6: Table S6).

### Mutation of *KAKU4* may fine-tune the chromatin states in the *Arabidopsis* genome

Our constructed network with the association proteins of lamin-like proteins revealed that KAKU4 is strongly associated with chromatin or epigenetic modifiers. Further global epigenome data analysis uncovered that KAKU4 had the greatest effect on H3K27me3, followed by H3K9me2, and the least effect on H3K4me3 in the *Arabidopsis* genome. In addition, multiple differential deposition peaks and their associated genes were identified between the *kaku4-2* mutant and WT. These results encourage us to study if and how the knockdown of *KAKU4* leads to a switch in chromatin states in the *Arabidopsis* genome. The discovery and characterization of the chromatin state transition caused by the knockdown of *KAKU4* may offer some important insights.

**Fig. 3:**
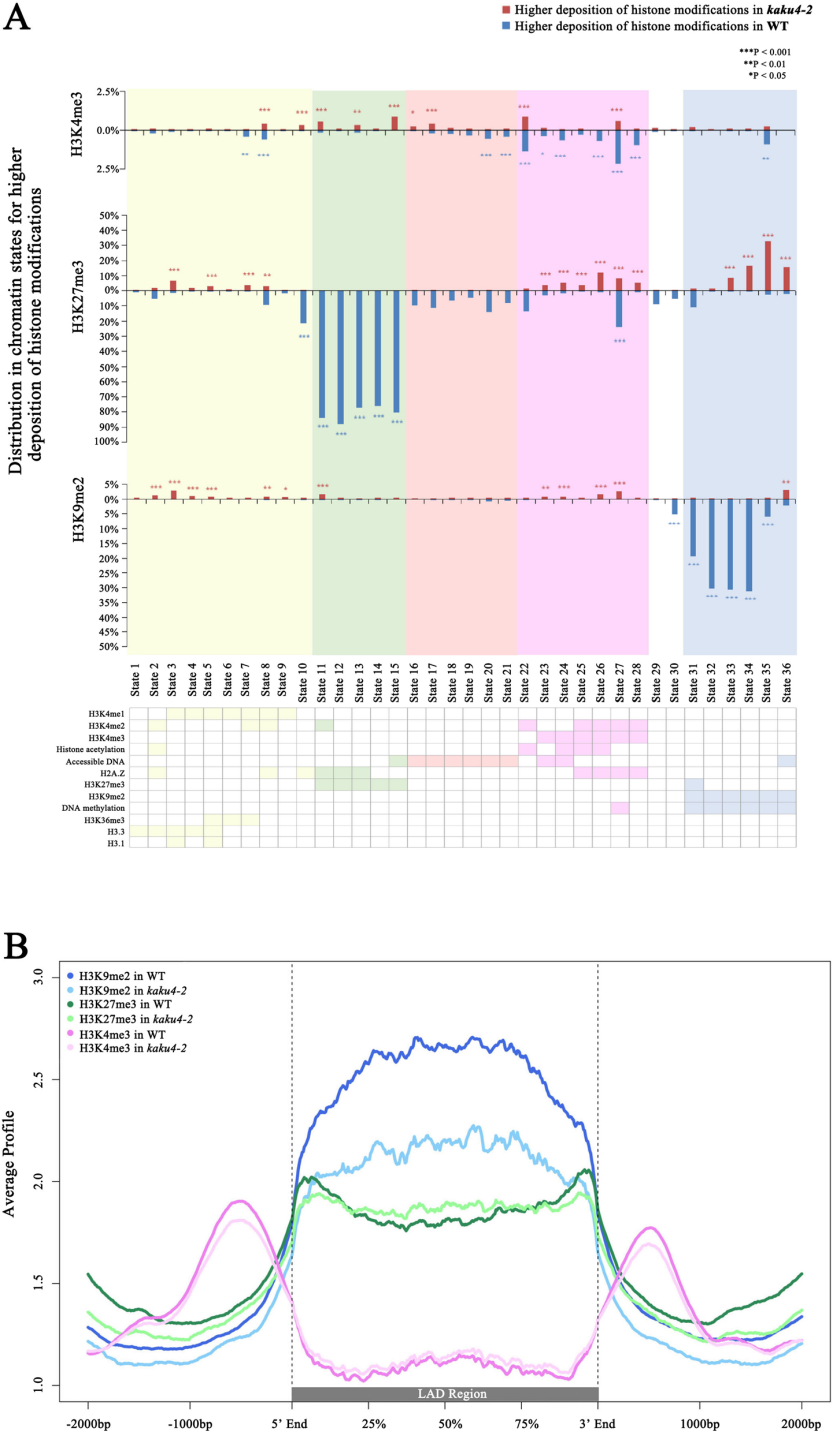
Chromatin state analysis for differential histone modification deposition between the *kaku4-2* mutant and WT. **A** Chromatin state analysis for the regions with higher deposition of H3K4me3, H3K27me3, and H3K9me2 in *kaku4-2* mutant and WT plants. The upper plot *X*-axis represents the 36 chromatin states, and the *Y*-axis represents the proportion of chromatin states enriched in the regions with higher H3K4me3, H3K27me3, and H3K9me2 deposition in the *kaku4-2* mutant and WT plants; red bars represent higher deposition of H3K4me3, H3K27me3, and H3K9me2 in *kaku4-2* mutant; the blue bars represent higher deposition of H3K4me3, H3K27me3, and H3K9me2 in the WT. ***P < 0.001, **P < 0.01, *P < 0.05. The lower panels represent a heatmap of the enriched epigenetic marks in each chromatin state. Color-filled indicates preferential epigenetic marks, while no color fill represents the absence or low signal of the indicated epigenetic mark. **B** Enrichment profiles of H3K4me3/H3K27me3/H3K9me2 deposition in PLADs. A profile was generated using the normalized sequencing density of H3K4me3/H3K27me3/H3K9me2 in *kaku4-2* mutant and WT. PLADs of different lengths are standardized to 3 kb. The 2 kb upstream and downstream regions of the PLAD are included

We adopted the information from the plant chromatin state database (PCSD) for this study. Our group constructed this database to improve the decoding of chromatin states with epigenomic data and discover causal functions hidden in plant chromatin [51]. In PCSD, the whole genome of *Arabidopsis thaliana* was defined as 36 chromatin states (290,553 segments) based on 216 published epigenetic data sets. Different chromatin states contain different epigenetic features: Chromatin states 16-28 (orange and purple background in Fig. 3A) are preferentially located in promoter and 5’ UTR regions, which are enriched in DHSs and active histone modification, such as H3K27ac and H3K4me3; chromatin states 11–15 (green background in Fig. 3A) are preferentially located in promoter and intergenic regions, which are enriched in LHP1 and repressive histone modification H3K27me3; chromatin states 31–36 (blue background in Fig. 3A) are preferentially located in TE regions, which are enriched in DNA methylation and repressive histone modification such as H3K9me2 [51]. Here, we used the bins of 36 *Arabidopsis* states as the reference and identified the regions with differential deposition of H3K4me3, H3K27me3, and H3K9me2 in the *kaku4-2* mutant and WT. The identified deposition regions were overlapped with state bins by more than 50%. The calculated state proportion in the total state is listed in Additional file 7: Table S7.

By exploring the chromatin states enriched in the regions with differential deposition of H3K4me3, H3K27me3, and H3K9me2 in the *kaku4-2* mutant and WT, the most significantly enriched chromatin states following *KAKU4* knockdown were in the H3K27me3-diminished regions, then by H3K9me2, with the least in H3K4me3 (the blue bars shown in Fig. 3A and Additional file 7: Table S7). We found that in the WT, the regions with higher H3K27me3 deposition were associated with states 11–15 and covered most bins in these states; the regions with higher H3K9me2 deposition were associated with states 31–35 and covered a large proportion of bins in these states. In the *kaku4-2* mutant, the regions with higher H3K27me3 deposition of were mainly associated with states 23-28 and 33-36, which were associated with H3K4me3 and H3K9me2, respectively; the regions with higher H3K9me2 deposition in the *kaku4-2* mutant were associated with states 2–9 and 23–27, which were associated with H3K4me1/2/3 (red bars shown in Fig. 3A and Additional file 7: Table S7). From another angle, Fig. 3A showed that in the states 11–15, corresponding to the typical H3K27me3-associated regions, the deposition of H3K27me3 was predominantly diminished in the *kaku4-2* mutant, while the H3K4me3 deposition was slightly increased; in the states 31–35, which are preferentially enriched in H3K9me2 and DNA methylation, the deposition of H3K9me2 was significantly diminished, whereas the H3K27me3 deposition was highly increased.

In addition, we analyzed the status change of chromatin states in the lamina-associated domain (LAD) regions. It has been reported that H3K9me2/H3K9me3 and H3K27me3 influence the frequency of NL-LAD interactions in mouse and human cells [42]. H3K9me2 is enriched in LADs and H3K27me3 is enriched near LAD borders [34]. In *Arabidopsis*, CRWN1 and CRWN4 were reported to be responsible for tethering specific pericentromeric chromatin regions to the nuclear periphery, and those non-accessible chromatin regions are termed plant lamina-associated domains [38]. Here, we conducted data mining and obtained PLAD information from the literature [38]. We examined the deposition profiles of H3K4me3, H3K27me3, and H3K9me2 in WT and *kaku4-2* mutant around these PLAD regions (Fig. 3B and Additional file 1: Fig S2A). In WT, H3K9me2 was significantly highly deposited in PLADs, while the deposition of H3K9me2 was lower in the *kaku4-2* mutant. In the regions near PLAD borders, H3K27me3 deposition was relatively lower in the *kaku4-2* mutant than in the WT. Meanwhile, in the middle regions of the PLADs, the deposition of H3K27me3 in the *kaku4-2* mutant was slightly higher than in the WT. The enrichment of H3K4me3 deposition regions was outside the PLAD regions, and there is no significant difference in the deposition of H3K4me3 between the WT and *kaku4-2* mutant (Fig. 3B and Additional file 1: Fig S2A). Meanwhile, we used the UCSC genome browser to show several typical regions near the PLAD (Additional file 1: Fig S2B). Our results indicated that the knockdown of *KAKU4* might cause a portion of the chromatin modified by H3K9me2 to be replaced by H3K27me3 modification.

Overall, the chromatin state analysis results showed that KAKU4 may fine-tune the chromatin states, and especially, the knockdown of *KAKU4* causes a transition between two types of repressive epigenetics marks, H3K9me2 and H3K27me3 in some specific PLAD genomic regions.

### KAKU4 plays an important role in H3K27me3-associated transcriptional repression

Global epigenome data analyses revealed that KAKU4 has the most significant effect on H3K27me3, followed by H3K9me2, and with the least effect on H3K4me3. Later, we performed GO enrichment analysis for the genes with differential deposition of H3K4me3, H3K27me3, and H3K9me2 in the WT and *kaku4-2* mutant. The results (Additional file 1: Fig S3) showed that the enriched GO terms for these genes, especially with higher deposition regions of H3K27me3 modification in the WT, covered broad biological processes, including organ development, aging, hormone pathways, etc. We further selected several marker genes and conducted ChIP-qPCR analyses to validate the ChIP-seq data indicating the H3K27me3 modification (Additional file 4: Table S13). PR2 is in response to pathogens. The metacaspases MC2 and MC1 antagonistically control programmed cell death in *Arabidopsis* [13]. SAG113/HAI1 functions as a negative regulator of osmotic stress and ABA signaling. WRKY58 is a negative regulator of defense responses and a direct target of NPR1 [87]. PHT3;2 encodes a mitochondrial phosphate transporter that is highly expressed in senescent leaves [85]. Overall, the ChIP-qPCR results validated the ChIP-seq results (Additional file 6: Table S6).

**Fig. 4:**
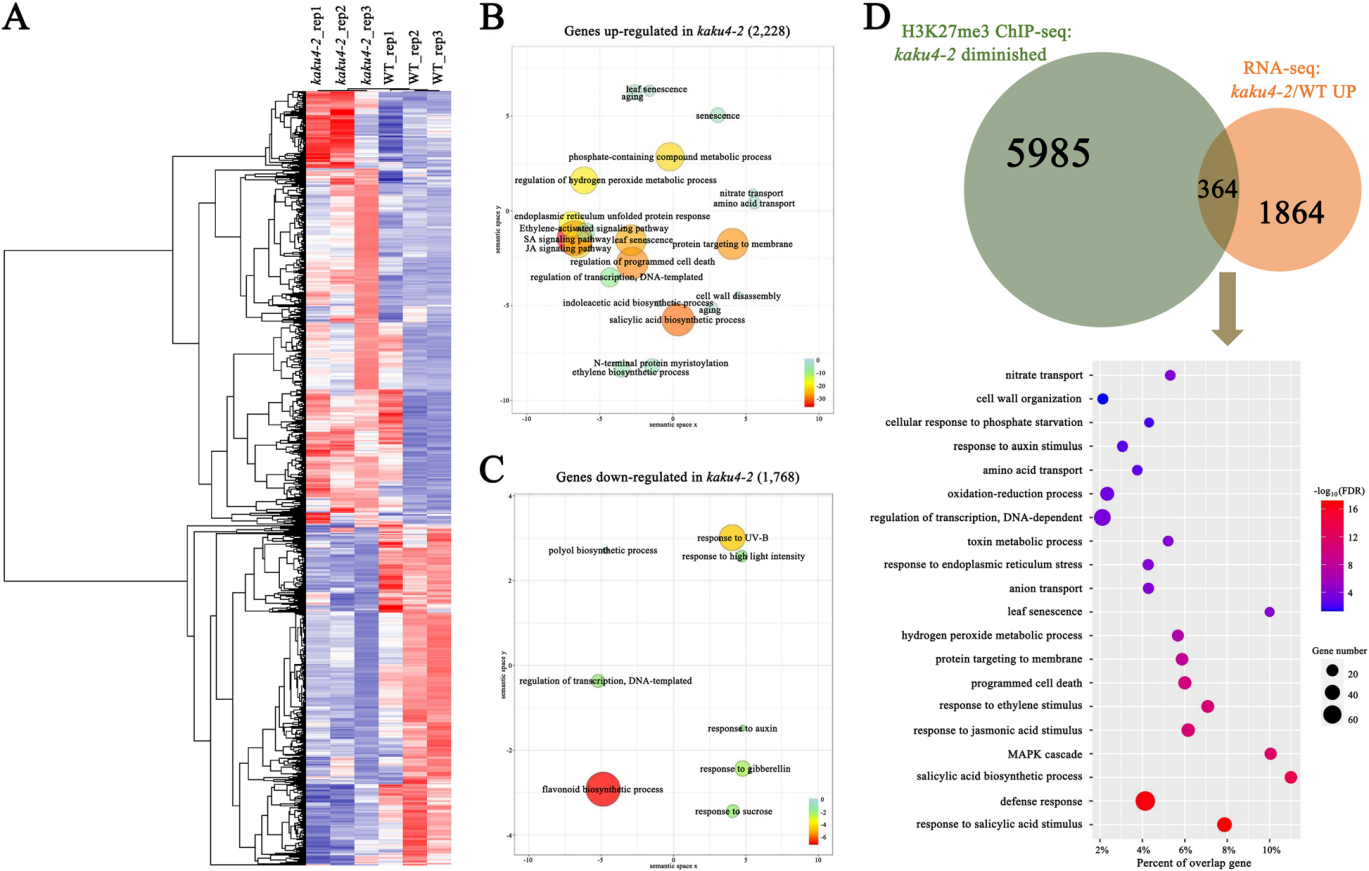
Combination analyses of epigenomic and transcriptomic data of the *kaku4-2* mutant and WT. **A** Heatmap showing relative expression levels of differentially expressed genes in different replicates of the *kaku4-2* mutant and WT. **B** GO enrichment analyses for genes upregulated in *kaku4-2* mutant by agriGOv2 and REVIGO. The scatter plot shows the cluster representatives in two-dimensional space derived by applying multidimensional scaling to a matrix of the significant GO terms with semantic similarities. The color and size of the bubble indicate the log_10_FDR (a legend in the bottom right-hand corner). The colors from red to green represent the significance level of the GO terms, from high to low. Fisher's exact test was conducted, and the *P*-value was adjusted using the Benjamini-Yekutieli method, FDR ≤ 0.05. **C** GO enrichment analyses for genes downregulated in *kaku4-2* mutant by agriGOv2 and REVIGO. **D** GO enrichment analyses for genes upregulated in the *kaku4-2* mutant and with higher deposition of H3K27me3 in the WT by agriGO and REVIGO

The knockdown of the *KAKU4* gene significantly modulated H3K27me3 modification in the *Arabidopsis* genome and may affect gene expression in some critical biological processes. Therefore, conducting combination analyses of epigenomic and transcriptomic data of the *kaku4-2* mutant and WT is necessary. We employed RNA-seq analysis to identify the genes regulated by KAKU4. Rosette leaves of the 30-day-old *kaku4-2* mutant and WT plants were sampled for RNA-seq, with three independent biological replicates (Additional file 4: Table S8). We performed differential expression analysis on RNA-seq data with the cutoff: |log_2_(fold change)| ≥ 0.6 and *P*-value ≤ 0.05. Compared with the WT, 2228 genes were upregulated in the *kaku4-2* mutant, and 1768 genes were downregulated in the *kaku4-2* mutant (Fig. 4A and Additional file 8: Table S9). The GO enrichment analysis for the differentially expressed genes (DEGs) is shown in Fig. 4B, C, and Additional file 9: Table S10, and the GSEA results are shown in Additional file 10: Table S11. In addition, several genes were selected for qRT-PCR validation of the RNA-seq results using additional biological replicate samples (Additional file 4: Table S14). The 11 selected marker genes were upregulated in the *kaku4-2* mutant. The expression levels of these genes validated the RNA-seq results very well (Additional file 8: Table S9). The selected genes included senescence marker genes SAG12 and SAG29, as well as SA pathway and plant immunity-related genes, such as PR1 and PR2 [2, 72]. LOX1 was involved in JA synthesis and has been reported to be related to leaf senescence [52].

Then, we conducted combination analyses of epigenomic and transcriptomic data between *kaku4-2* mutant and WT. We compared the DEGs with the differential deposition of histone modification (Additional file 1: Fig S4), and the DEGs with changes in H3K27me3 levels were much higher than those in H3K4me3 and H3K9me2. Thus, we mainly focused on the genes with differential deposition regions of H3K27me3 modification and DEGs between *kaku4-2* mutant and WT. We identified 364 genes with lower deposition of H3K27me3 and upregulation of gene expression in the *kaku4-2* mutant (Fig. 4D and Additional file 11: Table S12). Through GO enrichment analysis of the 364 genes, the enriched GO terms were mainly associated with hormone response, hydrogen peroxide metabolic process, programmed cell death, leaf senescence, etc. (Fig. 4D). We further measured the levels of several hormones in the rosette leaves of the *kaku4-2* mutant and WT plants (Additional file 4: Table S15). The content of SA, JA, and ABA in the rosette leaves of the *kaku4-2* mutant was 41-fold, 23-fold, and 7-fold higher, respectively, than in the WT. This experimental evidence partially supported the GO enrichment analysis results, suggesting that KAKU4 might affect the biosynthesis and signaling pathways of some phytohormones, such as SA, JA, and ABA.

## Discussion

The nuclear lamina (NL) links the nuclear membrane with chromosomes, which provides structural stability for the nucleus and plays a key role in regulating the three-dimensional chromatin structure and gene expression. Lamin is important in maintaining the overall round nuclear shape and the equilibrium of the epigenetic modifications [42]. The knowledge of nuclear lamina in plants is still limited compared to animals and humans. Hence, this study mainly focused on elucidating the mechanisms through which the putative nuclear lamina component protein KAKU4 regulates chromatin states and gene expression in *Arabidopsis* leaves. We conducted ChIP-seq technology to generate global epigenomic profiles of H3K4me3, H3K27me3, and H3K9me2 in *Arabidopsis* leaves for the mutant (*kaku4-2*) and wild-type (WT) plants, together with RNA-seq method to generate gene expression profiles. The comprehensive chromatin state-based analyses indicate that the knockdown of *KAKU4* has the strongest effect on H3K27me3, followed by H3K9me2, and the least impact on H3K4me3, leading to a significant change in chromatin states in the *Arabidopsis* genome. The knockdown of *KAKU4* caused a transition between two types of repressive epigenetics marks, H3K9me2 and H3K27me3, in some specific PLAD regions. We further employed combination analyses of epigenomic and transcriptomic data between *kaku4-2* mutant and WT, and the results suggested that KAKU4 may regulate some key biological processes in *Arabidopsis* leaves, such as hormone signaling pathways. Through multiomics conjoint analysis, we revealed the effect of lamin-like protein KAKU4 on chromatin state and gene expression.

While studying the potential functions of KAKU4, we find it interesting that lamin-like proteins in plants share some conserved functions with lamins in animals and humans. We compared the GO enrichment analysis results of the *Arabidopsis* lamin-like protein association network (Fig. 1A and Additional file 2: Table S1) and human lamin interaction network (Additional file 1: Fig S5) and found some similar enriched GO terms, including biological processes (BP) terms such as “histone modification,” “chromosome organization,” “gene silencing,” “cell cycle,” “DNA repair,” and “RNA processing,” and cell components (CC) terms such as “nuclear envelope,” “nuclear pore,” and “chromosome”. This indicated that *Arabidopsis* lamin-like proteins may play similar roles in humans and animals in regulating three-dimensional chromatin structure and gene expression.

In *Arabidopsis*, CRWN1 and CRWN4 are responsible for tethering specific pericentromeric chromatin regions to the nuclear periphery and the non-accessible chromatin regions are defined as PLADs, promoting transcriptional repression of the genes [38]. The fact that KAKU4 physically interacts with CRWN1 and CRWN4 [25] suggests that KAKU4 may also be associated with NL-chromatin interactions. Our results showed that the knockdown of *KAKU4* caused a portion of the chromatin modified by H3K9me2 to be replaced by H3K27me3 modification, and resulted in a chromatin state switch between two types of repressive epigenetics marks, H3K9me2 and H3K27me3, in some specific PLAD regions. These results presented here about lamin-like protein fine-tuning chromatin states seem very similar to those published using mammalian cells. In mammalian cells, large genome regions are enriched with repressive histone modification and are sparsely populated with active chromatin marks [5, 34, 36, 81]. The repressive epigenetic marks H3K9me2/H3K9me3 and H3K27me3 influence the frequency of the NL-LAD interactions in mouse and human cells [42]. H3K9 methylation is required for the integrity of LADs, and the loss of H3K9 methylation is compensated by the accumulation of H3K27me3 [47]. This research information related to nuclear lamina-chromatin interactions in mammalian cells provides us with a broad view and essential clues to study the potential functions of plant lamin-like proteins in regulating histone modification and chromatin state transition.

## Conclusions

In summary, we focused on performing a functional analysis of KAKU4 in *Arabidopsis* leaves. We revealed that KAKU4 is associated with chromatin/epigenetic modification regulators by analyzing the protein-protein association network of lamin-like proteins. Global epigenome data analyses uncovered that KAKU4 has the greatest effect on H3K27me3, followed by H3K9me2, and the least impact on H3K4me3. We discovered that the knockdown of *KAKU4* causes a significant change in chromatin states in the *Arabidopsis* genome. We found that the knockdown of *KAKU4* causes a transition between two types of repressive epigenetics marks, H3K9me2 and H3K27me3, in some specific PLAD regions. Furthermore, we conducted combination analyses of epigenomic and transcriptomic data for the *kaku4-2* mutant and WT leaves. Moreover, we found that KAKU4 potentially regulates some key biological processes by affecting H3K27me3 deposition, including oxidative stress, programmed cell death, and hormone signaling pathways. Further experimental results showed that the knockdown of the *KAKU4* gene caused hormone levels to be elevated, particularly SA, JA, and ABA. Specifically, the results of this study demonstrated the essential roles of KAKU4 in fine-tuning chromatin states and transcriptional regulation, affecting diverse biological processes in *Arabidopsis*. We hope our results on KAKU4 can enhance the understanding of lamin-like proteins affecting chromatin states and gene expression to a more systematic level in plants.

## Methods

### Plant materials and growth conditions

*Arabidopsis thaliana* (Col-0, *kaku4* mutant lines) seeds were surface sterilized and planted on half-strength Murashige and Skoog (MS) medium (1% (w/v) sucrose, pH 5.7 to 5.8 and 0.7% agar). After stratification at 4 °C for 3 days, the seeds were transferred to a conditioning chamber with a diurnal cycle of 16 h of light (22 °C) and 8 h of darkness (19 °C). After germination for 10 days, the seedlings were transferred to the soil.

### Identification of the *kaku4-2* T-DNA insertion mutant

The seeds of *kaku4-2* (SALK_076754) were obtained from the *Arabidopsis* Biological Resource Center (ABRC). Two consecutive PCR assays confirmed homozygous T-DNA insertion mutant plants. The first assay used two gene-specific primers: LP (SALK_076754-LP) and RP (SALK_076754-RP). The second assay used one gene-specific primer RP (SALK076754-RP) and one T-DNA-specific primer (LB). All primers used in this work are listed in Additional file 4: Table S16.

### Protein-protein association network construction

We collected protein-protein interaction/association pairs of lamin-like proteins from BioGRID [61] and literature [1, 4, 7, 8, 11, 28, 17, 21–25, 14, 29, 30, 35, 39–41, 48–50, 55, 57, 58, 62, 65, 69, 70, 75, 77–79, 91, 92, 95, 96]. We collected highly confident protein-protein association pairs (removed the protein-protein pairs that were only proved once by using mass spectrometry (MS) based experiments, such as IP-MS, clnip-MS, or affinity capture-MS) and divided the methods for proving protein interactions into four categories: protein-protein interaction validated by experiments (Affinity Capture-Western, BiFC, Co-IP, FRET, Luc, PCA, Reconstituted Complex, Two-hybrid), MS verification of single protein (Affinity Capture-MS, CF-MS, clnip-MS, CoIP-MS, IP-MS, PL-LFQMS), identification of large-scale yeast two-hybrid, identification of large-scale CF-MS. Then, the association network was visualized by Cytoscape v3.7.1 [67].

### Gene Ontology and gene set enrichment analysis

GO enrichment analysis was performed using agriGOv2 [80] and REVIGO [73]. Gene set enrichment analysis was conducted using PlantGSEA [90] and PlantGSAD [53].

### Western blot

Harvested leaves of *kaku4-2* mutant and WT were ground in liquid nitrogen and the powder was extracted with 2× SDS-PAGE loading buffer (P1040; Solarbio) at 100 °C for 10 min. The solution was centrifuged for 10 min at 12,000 rpm. Extracted proteins were separated on 10% SDS-PAGE gels and transferred to polyvinylidene difluoride (PVDF) membrane (Millipore, 0.22μm). Membranes were blocked in blocking buffer [5% milk dissolved in 1× TBST (Tris Buffered Saline with Tween 20)] at room temperature (24 °C) for 1 h. Membranes were incubated for 1.5 h at room temperature with antibodies against H3K4me3 (07-473; Millipore), H3K27me3 (07-449; Millipore), H3K9me2 (ab1220; Abcam), actin (AC009; ABclonal), and H3 (BE3015; EASYBIO) in blocking buffer. After washing five times in TBST for 3 min each, membranes were incubated for 1 h at room temperature with Anti-Rabbit VHH HRP (KTSM1322; AlpalifeBio) and Goat Anti-Mouse IgG, HRP Conjugated (CW0102S; CWBIO) diluted 1/2,000. Membranes were washed five times in TBST and incubated for 1 min in electrochemiluminescence (ECL) buffer (BE6706; EASYBIO).

### Chromatin immunoprecipitation and ChIP-seq analysis

A native chromatin immunoprecipitation (ChIP) was performed using anti-trimethyl-histone H3 (Lys 27) (H3K27me3), essentially as described with minor modification [59]. Approximately 20 g of the 4-week rosette leaves were ground to a fine powder in liquid nitrogen and re-suspended in TBS (3 mM CaCl_2_, 2 mM MgCl_2_, 10 mM Tris, pH 7.5, 0.1 mM PMSF, 2/5 tab of complete mini (Roche Applied Science, Indianapolis) with 0.5% Tween 40). Then, the nuclei were purified in a sucrose gradient and digested with 10 units of micrococcal nuclease (Sigma, St Louis) at 37 °C for 5 min. First, the nucleosome samples were incubated with pre-immune rabbit serum (1:100 dilution) and then with 4% protein A Sepharose (GH healthcare Bio-Sciences AB, Uppsala) for 4 h, before being centrifuged. The supernatant was incubated with anti-trimethyl-histone H3 (Lys 27) antibodies (Millipore, 07-449) at 4 °C overnight. An equal amount of pre-immune rabbit serum, which served as a nonspecific binding control in each ChIP experiment, was used in the control experiments. Then, the samples were incubated with 25% protein A Sepharose at 4 °C for 4 h. After centrifugation, the pellet (bound) fractions were subjected to a series of washes and the immune complexes were eluted from the washed beads using elution buffer (50 mM NaCl, 20 mM Tris-HCl at pH 7.5, 5 mM EDTA, 1% SDS). Immunoprecipitated DNA was extracted using phenol/chloroform and precipitated using ethanol. The ChIPed DNA was re-suspended in 100 μL TE buffer (pH 8.0).

ChIP-seq experiments using anti-trimethyl-histone H3 (Lys 9) antibodies (Abcam, ab1220) and anti-trimethyl-histone H3 (Lys 4) antibodies (Millipore, 07-473) were performed by Shanghai Jiayin Biotechnology Co., Ltd.

ChIP-seq libraries were constructed by Shanghai Jiayin Biotechnology Co., Ltd and sequenced using the Illumina system Novaseq 6000 with a 150 bp read length. All clean ChIP-seq reads were mapped to the *Arabidopsis* genome TAIR10 using BOWTIE2 software [46] with default parameters. The MACS program was used to shift the reads to identify peaks (bandwidth, 300 bp; model fold, 10, 30; *P* = 1.00e-5) [93]. The ChIP-seq data were visualized using the UCSC genome browser [43]. The distribution of peaks identified in the ChIP-Seq along the *Arabidopsis* genome was characterized using CEAS software [71]. After the positions of the peaks were determined, genes (including the 2-kb upstream and gene body regions) overlapping the peaks were considered to carry the epigenetic marks. All differential H3K4me3, H3K27me3, and H3K9me2 related peaks are listed in Additional file 5: Table S5 and were used for further analysis. All differential H3K4me3, H3K27me3, and H3K9me2 related genes are listed in Additional file 6: Table S6 and were used for further study.

### ChIP-qPCR

ECO Real-Time PCR system (Illumina) was used for quantitative real-time PCR analysis of prepared DNA in ChIP. The gene-specific primers used in ChIP-qRCR are shown in Additional file 4: Table S16. Experiments were repeated independently three times. To determine the relative fold enrichment (RFE) of modified histone-associated sequences in the bound fractions, 25S was used as a negative control. RFE was calculated as 2^−ΔΔCT^± standard deviation (SD), where ΔΔCT = ΔCT (positive control) − ΔCT (negative control).

### Chromatin state analysis

Using the bin of 36 chromatin states in *Arabidopsis* as the standard (state bin with chrC/M removed) from the PCSD (http://systemsbiology.cau.edu.cn/chromstates) [51], the regions with higher deposition of H3K4me3, H3K27me3, and H3K9me2 in *kaku4-2* mutant and WT were found to have more than 50% overlap with the state bin, calculating the percentage of calculated state in the total calculated state was conducted using the following formula:

$${{\text{Ratio}}}_{i}=\mathrm{count }({\text{simulated}}:\mathrm{ state}_{i})/\mathrm{count }(\mathrm{all\ state}_{i}) \ \ \ \ \ \ \ \ i=1-36$$

The chromatin state of selected Ratio > sum(count (simulated: state_1-36_))/sum(count (all state_1-36_)) is calculated for significance.

Hypgeomdist (count (simulated: state_*i*_), sum(count (simulated: state_1–36_)), count (all state_*i*_), sum(count (all state_1–36_))).

### RNA extraction and RNA-seq analysis

Approximately 150 mg of rosette leaves from the 30-day-old *Arabidopsis* plants were harvested and frozen in liquid nitrogen as independent biological samples. Total RNA was isolated using TRIZOL® reagent (Invitrogen, CA, USA) and purified using Qiagen RNeasy columns (Qiagen, Hilden, Germany) according to the manufacturer’s instructions. Sequencing libraries were constructed by the Beijing Genomics Institute and sequenced using an IlluminaHiSeq™ 2000 with 150-bp pair-end reads. RNA-seq data were generated from three biological replicates. The RNA-seq paired-end clean reads were aligned to the reference genome (TAIR10) by TopHat2 v2.0.9 software [44]. The mapping rate of each sample is shown in Additional file 4: Table S8. FPKM values were calculated using the Cufflinks [82], and the median value of FPKM in three RNA-seq replicates was used to calculate the log_2_FC. The differential expressed genes between WT and *kaku4-2* mutant were identified with the cutoff: |log_2_FoldChange| ≥ 0.6 and *P*-value ≤ 0.05.

### Quantitative real-time PCR

Reverse transcription was performed using Moloney Murine Leukaemia Virus (M-MLV; Invitrogen). A total of 10 μL samples containing 2 μg total RNA and 20 pmol of random hexamers (Invitrogen) were heated at 70 °C for 2 min and then chilled on ice for 2 min. Next, reaction buffer and M-MLV were added to a total volume of 20μL containing 200 units of M-MLV, 20 pmol random hexamers, 500 μM dNTPs, 50 mM Tris-HCl (pH 8.3), 3 mM MgCl_2_, 75 mM KCl, and 5 mM dithiothreitol. Then, the samples were incubated at 37 °C for 1.5 h [86].

Real-time PCR experiments were performed using gene-specific primers (Additional file 4: Table S16) on an ECO Real-Time PCR system (Illumina) according to the manufacturer’s protocol. Three biological repeats were performed using 8ng of cDNA with SYBR Green. *Arabidopsis* 18S rRNA was used for each sample as the internal control to normalize all data for the real-time RT-PCR experiments. The relative quantitation method (ΔΔCT) was employed to evaluate the quantitative variation among replicates. To compare gene-expression levels in the wild-type and *kaku4-2* mutant, we normalized the relative expression levels to the wild-type as a reference.

### Plant hormone content measurement

The rosette leaves of *Arabidopsis* plants grown in soil were harvested to measure hormone content. Rosette leaves were homogenized in liquid nitrogen, then added to 50 μL of the internal standard working solution and 500 μL of the extract solution, before being vortexed and shaken at 4 °C for 30 min. Next, 1 mL of the CHCl_3_ extract was added, followed by vortexing and shaking at 4 °C for 30 min. After centrifugation, the bottom layer of liquid was collected and dried with helium at room temperature. The samples were dissolved in 0.1 mL of MeOH and filtered through a 0.1-μM filter.

The samples were analyzed using a UPLC-HRMS system (UPLC, Waters ACQUITY UPLC i-Class, Milford, MA, USA; MS, Thermo Fisher Q-Exactive, Bremen, Germany) equipped with a heated electrospray ionization (HESI) source. A Poroshell 120 EC-C18 column (3.0 × 75 mm, 2.7 μm; Agilent, USA) was used to separate chromatographically at a flow rate of 0.4 mL min^−1^. The mobile phases consisted of 0.05% AA in water (phase A) and 0.05% AA in ACN (phase B) for the analysis of SA, ABA, JA, and IAA. To detect ZT and OPDA, 0.1% FA was added to water (phase A) and 0.1% FA to MeOH (phase B). The gradient program was set as follows: 90% A at 0 min to 60% A at 6.25 min, 10% A at 7.5 min and hold for 3 min, followed by a return to the initial condition. The column temperature was 35 °C. Both positive and negative ion modes were used in MS analysis. The HESI source parameters were set as follows: Spray voltage (+) at 3.5 kV, spray voltage (−) at 3 kV, capillary temperature at 320 °C, sheath gas flow rate at 30 arbitrary units, aux gas flow rate at 10 units, sweep gas flow rate at 5 units, gas heater temperature at 350 °C, and S-lens RF level at 55 %. Both the full MS scan (resolution = 70,000, AGC target = 3E6, maximum IT = 100 ms, scan range = 50–750 m/z) and the targeted MS2 scan (resolution = 17,500, AGC target = 2E5, maximum IT = 100 ms, isolation window = 2.0 m/z) were used for data acquisition.

### Supplementary Information

**Additional file 1:** **Fig. S1.** Western blot to detect H3K4me3/H3K27me3/H3K9me2 levels in *kaku4-2* mutant and WT. **Fig. S2.** Enrichment profiles of H3K4me3/H3K27me3/H3K9me2 deposition around the 5’ PLAD border and selected PLAD regions in the UCSC genome browser.**Fig. S3.** GO enrichment analysis for genes with higher deposition of H3K4me3, H3K27me3 and H3K9me2 in the *kaku4-2* mutant and WT.**Fig. S4.** Comparative analysis of H3K4me3 /H3K27me3 /H3K9me2-changed genes and differentially expressed genes between the *kaku4-2* mutant and WT. **Fig. S5.** Protein-protein interaction network analysis of human lamin proteins.

**Additional file 2:** **Table S1.** Lamin-like proteins and their associated proteins.

**Additional file 3:** **Table S2.** Gene set enrichment analysis (GSEA) for genes in the protein association network.

**Additional file 4:** **Table S3.** Statistical summary of ChIP sequencing libraries. **Table S4.** Number of peaks/genes with higher deposition of H3K4me3, H3K27me3 and H3K9me2 in the *kaku4-2* and WT. **Table S8.** Statistical summary of RNA sequencing libraries.** Table S13.** ChIP-qPCR of selected gene regions for H3K27me3 in the WT and *kaku4-2* leaves. **Table S14.** Real-time RT-PCR for selected genes in the WT and *kaku4-2* leaves. **Table S15**. Content of selected hormones in the WT and *kaku4-2* mutant leaves. **Table S16.** List of primers used in this work.

**Additional file 5:** **Table S5.** Peaks with higher deposition of H3K4me3, H3K27me3 and H3K9me2 in *kaku4-2* mutant and WT.

**Additional file 6:** **Table S6.** Genes with higher deposition of H3K4me3, H3K27me3 and H3K9me2 in *kaku4-2* mutant and WT.

**Additional file 7:** **Table S7.** The percentage of calculated state_i_ in all state_i_ (i: 1-36).

**Additional file 8:** **Table S9.** Differentially expressed genes between the *kaku4-2* mutant and WT.

**Additional file 9:** **Table S10.** The enriched GO terms for differentially expressed genes in the *kaku4-2* mutant and WT.

**Additional file 10:** **Table S11.** Gene set enrichment analysis (GSEA) for differentially expressed genes between the *kaku4-2* mutant and WT.

**Additional file 11:** **Table S12.** Genes with higher depositions of H3K27me3 in WT and up-regulated in *kaku4-2* mutant.

## Data Availability

All data generated or analyzed during this study are included in this published article, its supplementary information files, and publicly available repositories. All the raw datasets used in this manuscript were deposited in the NCBI Gene Expression Omnibus (GEO) with accession number GSE247174 (https://www.ncbi.nlm.nih.gov/geo/query/acc.cgi?acc= GSE247174).
