# Supplementary material for: Nuclear lamina component KAKU4 regulates chromatin states and transcriptional regulation in the Arabidopsis genome
Source: BMC Biol. 2024 Apr 12;22:80. doi: 10.1186/s12915-024-01882-5 (PMC11015597; doi:10.1186/s12915-024-01882-5)
Supplement: Supplementary file 1 — Additional file 1: Fig. S1. Western blot to detect H3K4me3/H3K27me3/H3K9me2 levels in kaku4-2 mutant and WT. Fig. S2. Enrichment profiles of H3K4me3/H3K27me3/H3K9me2 deposition around the 5’ PLAD border and selected PLAD regions in the UCSC genome browser.Fig. S3. GO enrichment analysis for genes with higher deposition of H3K4me3, H3K27me3 and H3K9me2 in the kaku4-2 mutant and WT.Fig. S4. Comparative analysis of H3K4me3 /H3K27me3 /H3K9me2-changed genes and differentially expressed genes between the kaku4-2 mutant and WT. Fig. S5. Protein-protein interaction network analysis of human lamin proteins. [file 12915_2024_1882_MOESM1_ESM.pdf]

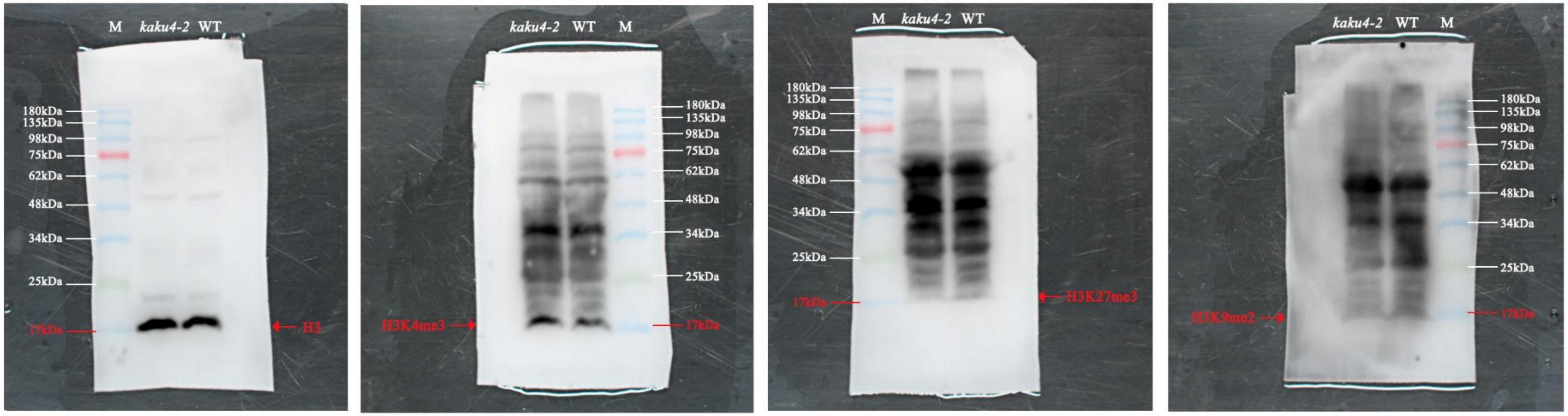

Anti-H3 Anti-H3K4me3 Anti-H3K27me3 Anti-H3K9me2

Supplementary Figure S1. Western blot to detect H3K4me3/H3K27me3/H3K9me2 levels in *kaku4* mutant and WT. Protein loading was assessed using an antibody that recognizes histone H3.

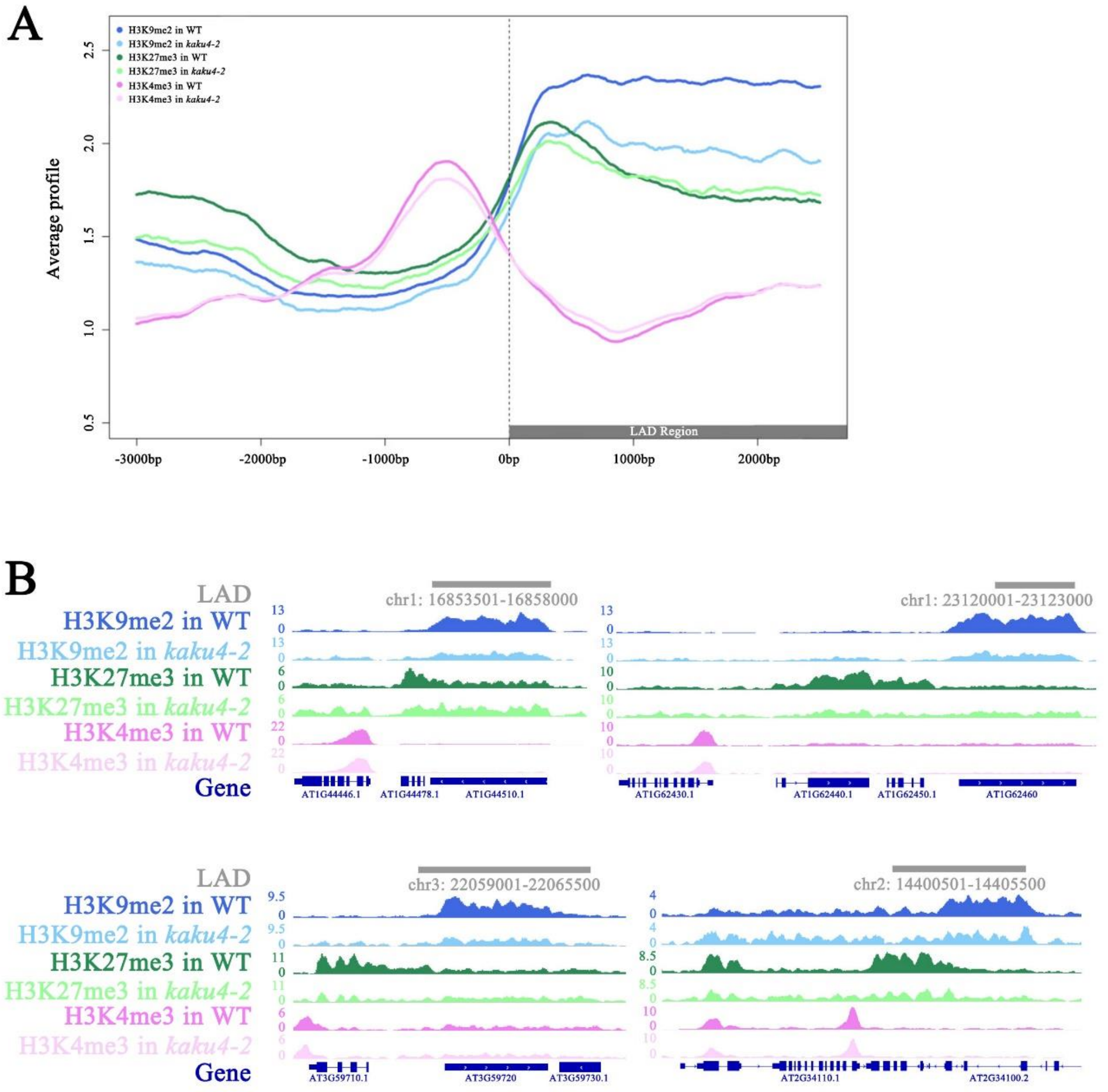

Supplementary Figure S2. Enrichment profiles of H3K4me3/H3K27me3/H3K9me2 deposition around the 5' PLAD border and selected PLAD regions in the UCSC genome browser.

A. Enrichment profiles of H3K4me3/H3K27me3/H3K9me2 deposition around the 5' PLAD border. A profile was generated using the normalized sequencing density of H3K4me3/H3K27me3/H3K9me2 in *kaku4-2* mutant and WT. Within 3 kb upstream/downstream from the 5' border of PLAD are included.

B. Selected PLAD regions in the UCSC genome browser. The brown colors represent the PLAD. The dark blue and light blue represent the H3K9me2 deposition in WT and *kaku4-2* mutant. The dark green and light green represent the H3K27me3 deposition in WT and *kaku4-2* mutant. The dark red and light red represent the H3K4me3 deposition in WT and *kaku4-2* mutant. The final dark blue column represents genes.

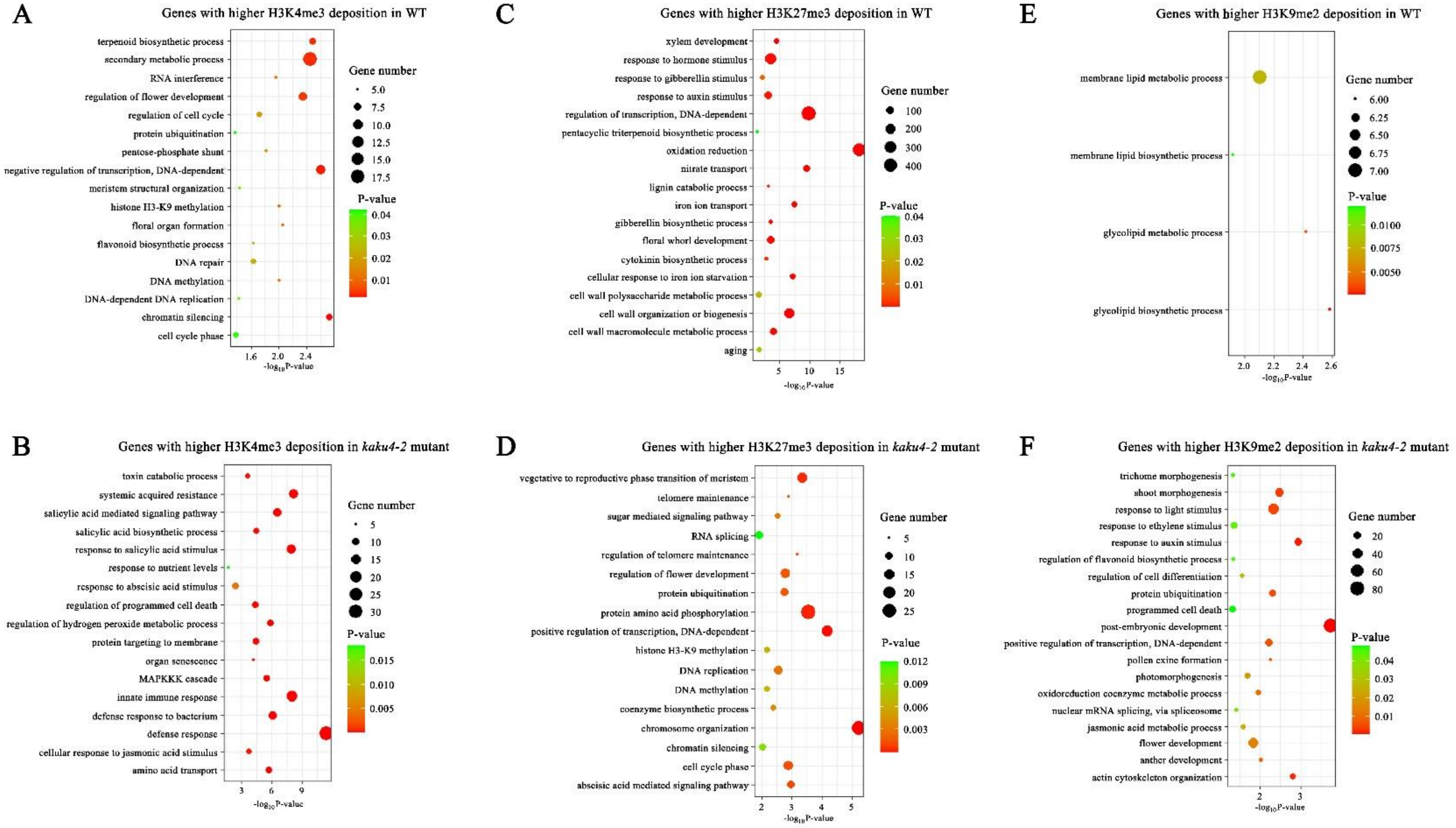
A
Genes with higher H3K4me3 deposition in WT
terpenoid biosynthetic process
secondary metabolic process
RNA interference
regulation of flower development
regulation of cell cycle
protein ubiquitination
pentose-phosphate shunt
negative regulation of transcription, DNA-dependent
meristem structural organization
histone H3-K9 methylation
floral organ formation
flavonoid biosynthetic process
DNA repair
DNA methylation
DNA-dependent DNA replication
chromatin silencing
cell cycle phase
Gene number
P-value
-log10P-value
C
Genes with higher H3K27me3 deposition in WT
xylem development
response to hormone stimulus
response to gibberellin stimulus
response to auxin stimulus
regulation of transcription, DNA-dependent
pentacyclic triterpenoid biosynthetic process
oxidation reduction
nitrate transport
lignin catabolic process
iron ion transport
gibberellin biosynthetic process
floral whorl development
cytokinin biosynthetic process
cellular response to iron ion starvation
cell wall polysaccharide metabolic process
cell wall organization or biogenesis
cell wall macromolecule metabolic process
aging
Gene number
P-value
-log10P-value
E
Genes with higher H3K9me2 deposition in WT
membrane lipid metabolic process
membrane lipid biosynthetic process
glycolipid metabolic process
glycolipid biosynthetic process
Gene number
P-value
-log10P-value
B
Genes with higher H3K4me3 deposition in kaku4-2 mutant
toxin catabolic process
systemic acquired resistance
salicylic acid mediated signaling pathway
salicylic acid biosynthetic process
response to salicylic acid stimulus
response to nutrient levels
response to abscisic acid stimulus
regulation of programmed cell death
regulation of hydrogen peroxide metabolic process
protein targeting to membrane
organ senescence
MAPKKK cascade
innate immune response
defense response to bacterium
defense response
cellular response to jasmonic acid stimulus
amino acid transport
Gene number
P-value
-log10P-value
D
Genes with higher H3K27me3 deposition in kaku4-2 mutant
vegetative to reproductive phase transition of meristem
telomere maintenance
sugar mediated signaling pathway
RNA splicing
regulation of telomere maintenance
regulation of flower development
protein ubiquitination
protein amino acid phosphorylation
positive regulation of transcription, DNA-dependent
histone H3-K9 methylation
DNA replication
DNA methylation
coenzyme biosynthetic process
chromosome organization
chromatin silencing
cell cycle phase
abscisic acid mediated signaling pathway
Gene number
P-value
-log10P-value
F
Genes with higher H3K9me2 deposition in kaku4-2 mutant
trichome morphogenesis
shoot morphogenesis
response to light stimulus
response to ethylene stimulus
response to auxin stimulus
regulation of flavonoid biosynthetic process
regulation of cell differentiation
protein ubiquitination
programmed cell death
post-embryonic development
positive regulation of transcription, DNA-dependent
pollen exine formation
photomorphogenesis
oxidoreduction coenzyme metabolic process
nuclear mRNA splicing, via spliceosome
jasmonic acid metabolic process
flower development
anther development
actin cytoskeleton organization
Gene number
P-value
-log10P-value

Supplementary Figure S3. GO enrichment analysis for genes with higher deposition of H3K4me3, H3K27me3 and H3K9me2 in the *kaku4-2* mutant and WT.

A. GO enrichment analysis for genes with a higher deposition of H3K4me3 in the WT. The colors of the circles indicate the $\log_{10}$FDR (a legend in the bottom right-hand corner), the colors from red to green represent the significance level of the GO terms from high to low, and similar GO terms are plotted closer together in the plot.

B. GO enrichment analysis for genes with a higher deposition of H3K4me3 in the *kaku4-2* mutant.

C. GO enrichment analysis for genes with a higher deposition of H3K27me3 in the WT.

D. GO enrichment analysis for genes with a higher deposition of H3K27me3 in the *kaku4-2* mutant.

E. GO enrichment analysis for genes with a higher deposition of H3K9me2 in the WT.

F. GO enrichment analysis for genes with a higher deposition of H3K9me2 in the *kaku4-2* mutant.

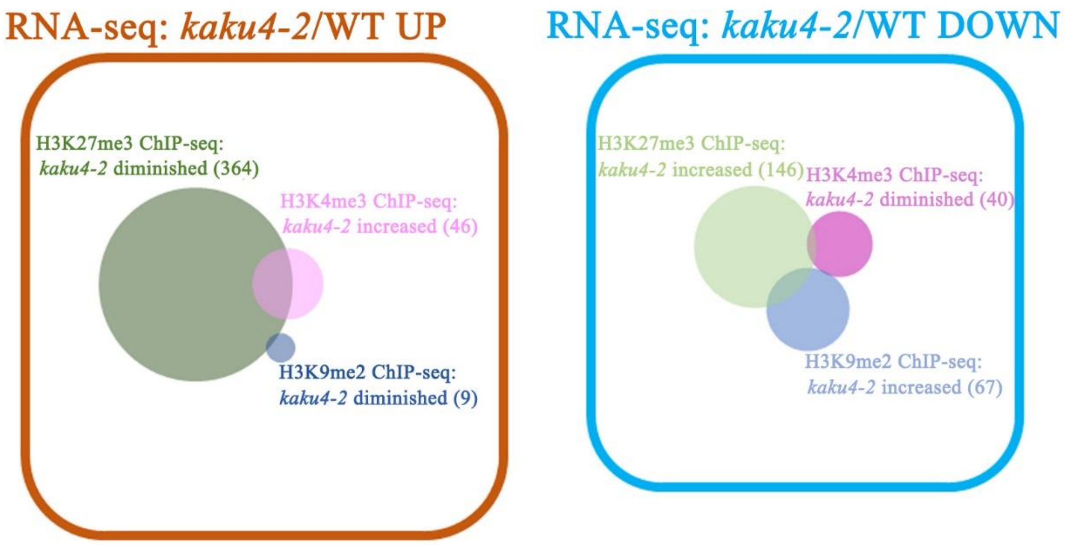

Supplementary Figure S4. Comparative analysis of H3K4me3 /H3K27me3 /H3K9me2-changed genes and differentially expressed genes between the *kaku4-2* mutant and WT.
Left represents the comparative analysis of the H3K4me3-increased, H3K27me3/H3K9me2-diminished genes and the up-regulated genes in the *kaku4-2* mutant; right represents the comparative analysis of the H3K4me3-diminished, H3K27me3/H3K9me2-increased genes and the down-regulated gene in the *kaku4-2* mutant.

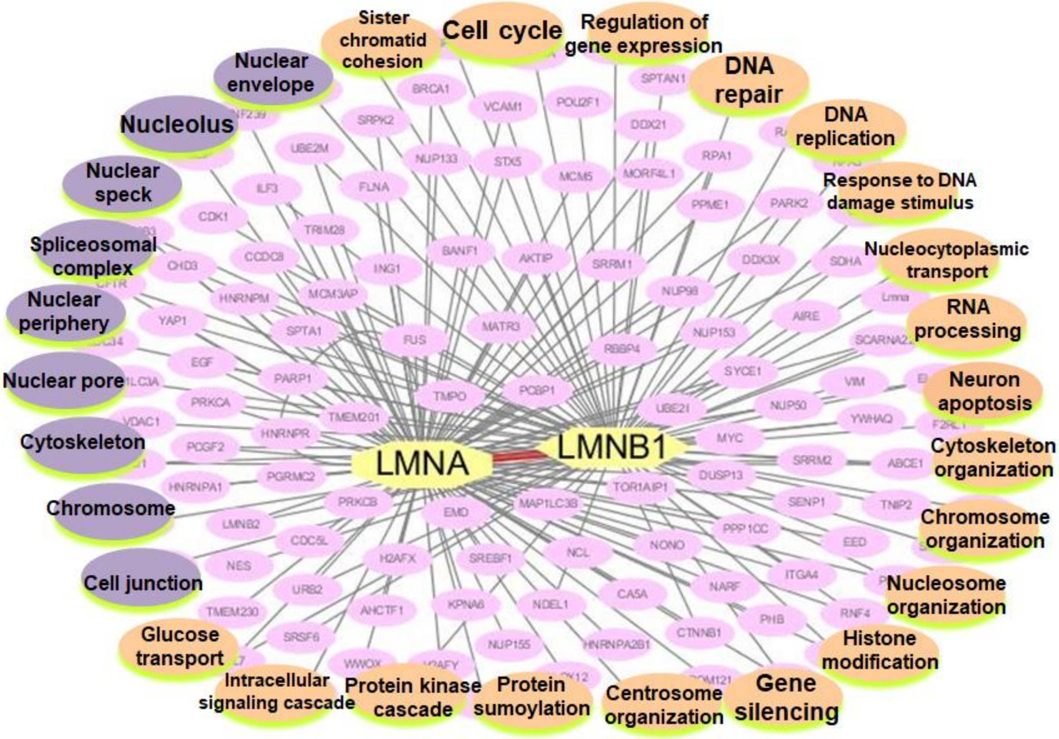

Supplementary Figure S5. Protein-protein interaction network analysis of human lamin proteins.

GO enrichment analysis of human lamin-interacting proteins; orange indicates terms of biological processes (BP), and grey indicates terms of cellular components (CC).
