# Supplementary material for: Nuclear lamina component KAKU4 regulates chromatin states and transcriptional regulation in the Arabidopsis genome
Source: BMC Biol. 2024 Apr 12;22:80. doi: 10.1186/s12915-024-01882-5 (PMC11015597; doi:10.1186/s12915-024-01882-5)
Supplement: Supplementary file 4 — Additional file 4: Table S3. Statistical summary of ChIP sequencing libraries. Table S4. Number of peaks/genes with higher deposition of H3K4me3, H3K27me3 and H3K9me2 in the kaku4-2 and WT. Table S8. Statistical summary of RNA sequencing libraries. Table S13. ChIP-qPCR of selected gene regions for H3K27me3 in the WT and kaku4-2 leaves. Table S14. Real-time RT-PCR for selected genes in the WT and kaku4-2 leaves. Table S15. Content of selected hormones in the WT and kaku4-2 mutant leaves. Table S16. List of primers used in this work. [file 12915_2024_1882_MOESM4_ESM.docx]

**Supplementary Table S3 | Statistical summary of ChIP sequencing libraries.**

| **Type** | **Sample** | **Reads mapping to genome** | **Peaks** | **Overlapped peaks** |
| --- | --- | --- | --- | --- |
| H3K4me3 | *kaku4-2* | 39,985,886 | 13875 | 13589 (97.94%) |
|  | WT | 44,692,549 | 14056 | 13601 (96.76%) |
| H3K27me3 | *kaku4-2* | 20,674,541 | 3141 | 2123 (67.59%) |
|  | WT | 34,623,553 | 5424 | 995 (18.34%) |
| H3K9me2 | *kaku4-2* | 17,750,830 | 2947 | 2173 (73.74%) |
|  | WT | 54,247,989 | 2008 | 721 (35.91%) |

**Supplementary Table S4 | Number of peaks/genes with higher deposition of H3K4me3, H3K27me3 and H3K9me2 in the *kaku4-2* and WT.**

| **Type** | **Comparison** | **NO. of Peaks** | **NO. of Genes** |
| --- | --- | --- | --- |
| H3K4me3 | Higher deposition of H3K4me3 in *kaku4-2* | 120 | 124 |
|  | Higher deposition of H3K4me3 in WT | 166 | 223 |
| H3K27me3 | Higher deposition of H3K27me3 in *kaku4-2* | 2417 | 2901 |
|  | Higher deposition of H3K27me3 in WT | 5510 | 6349 |
| H3K9me2 | Higher deposition of H3K9me2 in *kaku4-2* | 595 | 770 |
|  | Higher deposition of H3K9me2 in WT | 1180 | 1265 |

**Supplementary Table S8 | Statistical summary of RNA sequencing libraries.**

| **Sample** | **Total reads** | **Mapped Reads** | **Mapping rate** | **Properly paired** |
| --- | --- | --- | --- | --- |
| *kaku4-2* rep1 | 46,973,278 | 43,579,169 | 92.80% | 88.70% |
| *kaku4-2* rep2 | 46,867,658 | 43,416,110 | 92.60% | 88.50% |
| *kaku4-2* rep3 | 47,207,868 | 43,776,444 | 92.70% | 87.80% |
| WT rep1 | 47,155,550 | 43,775,202 | 92.80% | 88.20% |
| WT rep2 | 47,067,472 | 43,657,059 | 92.80% | 88.70% |
| WT rep3 | 47,106,766 | 43,732,432 | 92.80% | 88.60% |

**Supplementary Table S13 | ChIP-qPCR of selected gene regions for H3K27me3 in WT and *kaku4-2* leaves**

| **Locus ID** | **Gene Name** | **WT** | | | ***kaku4-2*** | | | **Fold Change** |
| --- | --- | --- | --- | --- | --- | --- | --- | --- |
|  |  | **rep1** | **rep2** | **rep3** | **rep1** | **rep2** | **rep3** | **(*kaku4-2*/ WT)** |
| AT4G25110 | MC2 | 0.97 | 1.02 | 1.02 | 0.49 | 0.48 | 0.52 | 0.50±0.003 |
| AT3G48850 | PHT3;2 | 0.83 | 1.00 | 1.21 | 0.65 | 0.66 | 0.60 | 0.63±0.003 |
| AT3G57260 | PR2 | 1.05 | 1.01 | 0.94 | 0.61 | 0.60 | 0.72 | 0.64±0.007 |
| AT5G59220 | SAG113 | 1.09 | 0.94 | 0.98 | 0.66 | 0.68 | 0.69 | 0.68±0.002 |
| AT3G01080 | WRKY58 | 0.99 | 1.15 | 0.88 | 0.55 | 0.74 | 0.79 | 0.68±0.012 |

**Supplementary Table S14 | Real-time RT-PCR for selected genes in WT and *kaku4-2* leaves**

| **Locus ID** | **Gene Name** | **WT** | | | ***kaku4-2*** | | | **Fold Change** |
| --- | --- | --- | --- | --- | --- | --- | --- | --- |
|  |  | **rep1** | **rep2** | **rep3** | **rep1** | **rep2** | **rep3** | **(*kaku4-2*/ WT)** |
| AT2G29350 | SAG13 | 1.22 | 1.77 | 0.46 | 35.42 | 1.43 | 2.65 | 5.11±0.825 |
| AT5G13170 | SAG29 | 1.17 | 0.88 | 0.97 | 31.83 | 27.86 | 27.77 | 29.09±0.592 |
| AT3G60140 | DIN2/SRG2 | 1.02 | 1.29 | 0.76 | 1.31 | 1.21 | 3.25 | 1.73±0.037 |
| AT2G14610 | PR1 | 1.07 | 1.12 | 0.83 | 2.00 | 2.27 | 2.26 | 2.17±0.014 |
| AT3G57260 | PR2/BGL2 | 0.78 | 1.28 | N/A | 2.91 | 3.45 | 3.96 | 3.41±0.081 |
| AT3G26830 | PAD3 | 0.82 | 1.22 | N/A | 2.23 | 1.13 | 1.46 | 1.55±0.018 |
| AT1G55020 | LOX1 | 0.87 | 1.15 | 0.99 | 2.35 | 7.25 | 5.59 | 4.57±0.336 |
| AT5G43580 | UPI | 1.35 | 0.69 | 1.07 | 4.66 | 8.34 | 18.47 | 8.96±1.112 |
| AT2G38470 | WRKY33 | 0.91 | 1.09 | 1.01 | 2.31 | 2.76 | 2.50 | 2.52±0.025 |
| AT3G15500 | NAC055/NAC3 | 1.04 | 1.02 | 0.94 | 4.08 | 3.34 | 4.78 | 4.03±0.096 |
| AT1G18570 | MYB51 | 1.23 | 0.91 | 0.89 | 1.61 | 1.95 | N/A | 1.77±0.012 |
| AT4G31430 | KAKU4 | 1.36 | 0.75 | 0.98 | 0.23 | 0.20 | 0.26 | 0.23±0.004 |

**Supplementary Table S15 | Content of selected hormones in the WT and *kaku4-2* mutant leaves**

| **Hormone** | **WT** | | ***kaku4-2*** | |
| --- | --- | --- | --- | --- |
|  | **rep1** | **rep2** | **rep1** | **rep2** |
| **SA** (ng/g) | 102.68 | 105.89 | 4317.23 | 4279.70 |
| **JA** (ng/g) | 120.59 | 92.37 | 2555.65 | 2473.01 |
| **ABA** (ng/g) | 10.73 | 11.99 | 79.51 | 88.31 |
| **IAA** (ng/g) | 3.36 | 5.66 | 3.89 | 6.38 |

**Supplementary Table S16 | List of primers used in this work**

| **Primer name** | **Sequence (5'-3')** | **Application** |
| --- | --- | --- |
| SALK_076754-LP | ACCAAGCATTCAACGACTCAG | Genotyping *kaku4* |
| SALK_076754-RP | GGATGAGGAAAAGTTTCCAGC | Genotyping *kaku4* |
| LB | GCGTGGACCGCTTGCTGCAACT | Genotyping *kaku4* |
| kaku4Q-FP | GCTCCTCAAAGTGGAACTGC | Real-time RT-PCR |
| kaku4Q-RP | CCAAGACAGCATTTGTGGTG | Real-time RT-PCR |
| SAG13_QF | TTAAAAGCCTCTGGCTCAGG | Real-time RT-PCR |
| SAG13_QR | TGGATCCAACATTGACATGC | Real-time RT-PCR |
| SAG29_QF | CTTCCTCACTATAAGCGCCG | Real-time RT-PCR |
| SAG29_QR | CAGCCCTAGTACGAATCCCA | Real-time RT-PCR |
| DIN2_QF | TCTTTCATGGCAATGAACCA | Real-time RT-PCR |
| DIN2_QR | GCCTTGCAAAACACCAAAAT | Real-time RT-PCR |
| PR1_QF | CATACACTCTGGTGGGCCTT | Real-time RT-PCR |
| PR1_QR | TCGCTAACCCACATGTTCAC | Real-time RT-PCR |
| PR2_QF | GGAGGCGAGACGTTCAAGAT | Real-time RT-PCR |
| PR2_QR | ATGCTAGGCGATACCTTGCC | Real-time RT-PCR |
| PAD3_QF | TGCTCCCAAGACAGACAATG | Real-time RT-PCR |
| PAD3_QR | GTTTTGGATCACGACCCATC | Real-time RT-PCR |
| LOX1_QF | TGGAGACAAATTTGGAGCAG | Real-time RT-PCR |
| LOX1_QR | AAAGACCGGGTGAAGAACAC | Real-time RT-PCR |
| UPI_QF | GGAACAGTTGTTCCGGAGAT | Real-time RT-PCR |
| UPI_QR | AATTTGAACAACGATTCCGC | Real-time RT-PCR |
| WRKY33_QF | GGAGAGAGCATCACACGACA | Real-time RT-PCR |
| WRKY33_QR | GTGCTCTGTTTGTGGCGTAA | Real-time RT-PCR |
| NAC3_QF | TTCTCGAGTCGTTGCATGAG | Real-time RT-PCR |
| NAC3_QR | ATCCCCTCAGTTTGTTGCTG | Real-time RT-PCR |
| MYB51_QF | CGTTGCAACAACACCAGTTT | Real-time RT-PCR |
| MYB51_QR | GGAGGAATCAGAGAACGTGG | Real-time RT-PCR |
| PR2-FP | GGAGGCGAGACGTTCAAGAT | ChIP-qPCR |
| PR2-RP | ATGCTAGGCGATACCTTGCC | ChIP-qPCR |
| PHT3;2-FP | CCGGCGAGTACATTTCCACT | ChIP-qPCR |
| PHT3;2-RP | GTCTTTTTCAGGCGACGACC | ChIP-qPCR |
| SAG113-FP | CGCCGGAAACAAACTTAAACCT | ChIP-qPCR |
| SAG113-RP | GGCTGAGATTTGTTACGAGAACG | ChIP-qPCR |
| MC2-FP | GACTATCACCGCTCGCTTCT | ChIP-qPCR |
| MC2-RP | ACCTTCTCCGTACACTCACG | ChIP-qPCR |
| WRKY58-FP | TAGTCTAAGCCCAGGGCCAC | ChIP-qPCR |
| WRKY58-RP | GGAGACACCGACATAGTTCCG | ChIP-qPCR |
